# A Technology-Assisted, Brief Mind-Body Intervention to Improve the Waiting Room Experience for Chemotherapy Patients: Randomized Quality Improvement Study

**DOI:** 10.2196/13217

**Published:** 2019-11-07

**Authors:** Ting Bao, Gary Deng, Lauren A DeMarzo, W Iris Zhi, Janice L DeRito, Victoria Blinder, Connie Chen, Qing S Li, Jamie Green, Eva Pendleton, Jun J Mao

**Affiliations:** 1 Integrative Medicine Service Department of Medicine Memorial Sloan Kettering Cancer Center New York, NY United States

**Keywords:** mobile app, acupressure, meditation, symptom relief, chemotherapy

## Abstract

**Background:**

Patients waiting for chemotherapy can experience stress, anxiety, nausea, and pain. Acupressure and meditation have been shown to control such symptoms.

**Objective:**

This study aimed to evaluate the feasibility and effectiveness of an integrative medicine app to educate patients about these self-care tools in chemotherapy waiting rooms.

**Methods:**

We screened and enrolled cancer patients in chemotherapy waiting rooms at two Memorial Sloan Kettering Cancer Center locations. Patients were randomly assigned into an intervention arm in which subjects watched acupressure and meditation instructional videos or a control arm in which they watched a time- and attention-matched integrative oncology lecture video. Before and after watching the videos, we asked the patients to rate four key symptoms: stress, anxiety, nausea, and pain. We performed the analysis of covariance to detect differences between the two arms postintervention while controlling for baseline symptoms.

**Results:**

A total of 223 patients were enrolled in the study: 113 patients were enrolled in the intervention arm and 110 patients were enrolled in the control arm. In both groups, patients showed significant reductions in stress and anxiety from baseline (all *P*<.05), with the treatment arm reporting greater stress and anxiety reduction than the control arm (1.64 vs 1.15 in stress reduction; *P*=.01 and 1.39 vs 0.78 in anxiety reduction; *P*=.002). The majority of patients reported that the videos helped them pass time and that they would watch the videos again.

**Conclusions:**

An integrative medicine self-care app in the waiting room improved patients’ experiences and reduced anxiety and stress. Future research could focus on expanding this platform to other settings to improve patients’ overall treatment experiences.

## Introduction

Ambulatory parenteral chemotherapy is a common mainstream cancer treatment. Long wait times for chemotherapy can cause patient dissatisfaction and anticipatory nausea [1,2]. At the time of this study, a commonly cited complaint among patients was a long waiting time from check-in to receiving chemotherapy at the Memorial Sloan Kettering Cancer Center’s (MSK) Rockefeller Outpatient Pavilion and Breast and Imaging Center. Long delays in chemotherapy waiting rooms can trigger patients to feel stress, anxiety, pain, and anticipatory nausea. These symptoms can cause adverse treatment experiences and incur health care cost if intervention is needed.

Integrative therapies such as acupressure and mindfulness meditation are used by individuals undergoing cancer treatments to alleviate symptoms such as pain, nausea, and anxiety. Acupressure is a therapy achieved by pressing along acupoints, which are histologically distinct cutaneous areas with high electrical conductivity, throughout the body [3]. Stimulating these acupoints releases endorphins and various neurotransmitters, such as serotonin and norepinephrine, which consequently provide symptomatic relief [4-6]. Acupressure provides a nonpharmacological, noninvasive approach for patients to effectively manage emotional and physical conditioned responses they experience in the waiting room [7]. The efficacy of acupressure in reducing chemotherapy-induced nausea and vomiting has been suggested through numerous clinical trials and systematic reviews [8-10]. Among them, the subgroup analysis of a meta-analysis of 11 randomized controlled trials (N=1247) on acupuncture point stimulation showed that acupressure significantly reduced mean acute chemo-induced nausea severity and most severe acute nausea [8,9]. Similarly, mindfulness meditation has been extensively studied and shown to reduce anxiety and stress [11-14]. It is a mind-body technique that places intentional focus on the present state of the body to achieve relaxation. Both acupressure and mindfulness meditation are accessible, low-cost therapeutic modalities that can be self-administered by patients with no adverse side effects.

Although useful, these integrative modalities are not readily available in the outpatient setting. Written instructions on acupressure are available in MSK’s Web-based patient and caregiver education library, but patients are either not aware of or do not have access to them. Podcasts on guided imagery and relaxation techniques are also available on MSK’s website but are rarely used because of inconvenience. Previous integrative medicine classes adjacent to chemotherapy waiting rooms had low attendance rates as patients were fearful of missing their appointments.

A potential solution to effectively deliver therapeutic modalities directly to patients is through technological apps. Technological apps have become increasingly prevalent in the health care setting because of their ease of accessibility and enhancement of patient care through personalization. Today, technological apps are used to monitor and manage symptoms in patients with diabetes, cancer, inflammatory bowel disease, depression, and anxiety [15]. Through these apps, patients can access medical guidance more easily and also develop an increased sense of agency over their symptoms.

Researchers have also studied the use of apps to manage and monitor symptoms as well as to improve medication adherence in patients with chronic conditions [16]. Studies have also examined the effectiveness of psycho-educational apps in providing self-guided mental health interventions for patients outside the clinical setting [17], and studies have investigated how to efficiently use apps in obstetrics and gynecology waiting rooms to educate patients on topics such as contraception [18]. However, to our knowledge, this is the first study to examine the effects of using an app to deliver integrative therapies and education to alleviate adverse symptoms induced by long wait times in chemotherapy waiting rooms. As such, we conducted a quality improvement (QI) study to evaluate the feasibility and effects of introducing an integrative medicine app at MSK, delivering self-administered therapeutic techniques to improve the overall treatment experience. A QI study allows us to directly assess whether a new process, such as this one, provides direct benefit to patient outcomes and overall quality of care.

## Methods

### Participants

We obtained an MSK Institutional Review Board waiver as the study represented minimal risk, and we did not collect any personal health information. Patients scheduled to receive chemotherapy at 2 locations at MSK (Rockefeller Outpatient Pavilion and Breast and Imaging Center) were consecutively screened as they checked in for their appointments. Patients who were receiving chemotherapy for the first time and non-English speaking patients were excluded.

Without previous knowledge of the assignment, an MSK staff member would approach patients with a sealed envelope containing assignment group A or B. Patients were asked if they would like to watch a video and participate in a survey. Once patients agreed, a staff member would then open the sealed envelope to reveal the patient’s assigned group (video A or B). Study staff then selected the appropriate video and briefed patients on how to use the app on a tablet in the waiting room. Patients proceeded to watch either the intervention or control video (both 15 min long).

### App

All integrative medicine education videos and surveys were run through a third-party health care app, Tonic Health. MSK Compliance and Information Security conducted a controls security assessment and hands-on penetration test to confirm Tonic Health as a secure app that complied with the MSK Security policy.

### Intervention and Control

Patients randomized to the intervention arm watched videos instructing them to perform acupressure at 3 acupoints known to reduce anxiety, stress, and nausea: pericardium-6 (located 3 finger-breadths below the wrist on the inner forearm in between the 2 tendons), Yin Tang (located midway between the medial ends of 2 eyebrows), and stomach-36 (located 4 finger-widths down 1 finger lateral from the bottom of the knee cap). In addition to the acupressure session, intervention patients also viewed a guided meditation video that included a series of breathing and visualization exercises accompanied by gentle music. The script was developed by an MSK mind-body therapist, MSK clinical staff recorded all content, and the MSK Video and Conference Services department edited the content. Patients randomized to the control arm were instructed to watch a 15-min lecture by an integrative medicine physician introducing integrative medicine modalities for cancer patients.

### Outcomes

All patients were asked 4 questions to rate the levels of nausea, stress, headaches/generalized pain, and anxiety they were experiencing at the start and end of the video. They assessed the degree of each of their symptoms using a 0-10 Likert numerical rating scale (with 0 indicating no symptoms and 10 indicating worst symptoms imaginable). This scale has been used previously in clinical trials to effectively assess symptoms such as pain, stress, and anxiety [19-23]. At the end of the session, patients provided qualitative feedback on the study by rating their satisfaction through the following 3 questions: (1) Did you find the videos helpful?, (2) Did the videos help you pass time?, and (3) Are you interested in watching MSK self-care videos in the future? These questions were then followed by an open-field comments section for patients to provide testimonials regarding their experiences.

### Statistical Analysis

The means and standard deviations for symptom scores were calculated in both the intervention and control arms. Paired *t* tests were used to evaluate changes in symptom score before and after watching the videos in each treatment arm. The difference in symptom scores between treatments was assessed with an analysis of covariance model, with the postvideo symptom score as the outcome and the treatment group and baseline symptom score as covariates. All analyses were performed using Stata 12 (StataCorp).

## Results

### Participants

From October 19, 2016, to December 29, 2016, we approached 363 patients when they arrived at 2 chemotherapy waiting rooms at MSK. Among these, 61.4% (223/363) agreed to participate in the study, and they were subsequently randomized into the intervention arm (n=113) or the control arm (n=110). Within the intervention arm, 103 out of 113 patients (91.1%) completed the intervention video and survey. Within the control arm, 102 out of 110 patients (92.7%) completed the education video and survey. Of the 363 patients approached, 140 patients declined to participate in the study, as they were either called in for their appointment or chose to engage in other activities during the waiting period. Of the 223 participants, 9 patients in the intervention arm were called into their appointment before completing the video, and 1 patient stated that the video made her more anxious. In the control group, 7 patients were called into their appointment before completing the video, and 1 patient opted to spend time with family instead. There were no significant differences in baseline measures between dropouts and patients who completed the study.

### Symptoms

Baseline symptoms were similar between the intervention and control arms. After watching the intervention self-care videos, patients’ stress, pain, and anxiety were significantly reduced compared with baseline (stress from 3.6 to 1.9, pain from 1.8 to 1.5, and anxiety from 3.1 to 1.7; all *P*<.05), without significant changes in nausea 2A). After watching the control video, patients’ stress and anxiety were also significantly reduced (stress from 3.6 to 2.5 and anxiety from 3.0 to 2.2; both *P*<.001), without significant changes in nausea and pain. Compared with the control arm, the intervention arm showed a significantly greater reduction in stress and anxiety (1.64 vs 1.15 in stress reduction; *P*=.01 and 1.39 vs 0.78 in anxiety reduction; *P*=.002 (Table 1).

**Table 1:** Symptoms changes in both the intervention and control groups.

Symptoms	Intervention group, mean (SD)		Control group, mean (SD)		Between group *P* value
	Pre	Post	Pre	Post	
Stress	3.5 (2.5)	1.9 (2.0)	3.6 (2.6)	2.5 (2.3)	.01^a^
Pain	1.8 (2.1)	1.5 (1.9)	1.7 (2.2)	1.5 (2.2)	.55
Nausea	0.9 (1.9)	0.8 (1.9)	0.9 (1.9)	0.7 (1.4)	.32
Anxiety	3.2 (2.7)	1.7 (2.2)	3.1 (2.7)	2.2 (2.2)	.002^a^

^a^Statistically significant value: <.05.

### Patient Experience

We surveyed patients in both the intervention and control groups regarding their experiences using these videos (Table 2).

In the intervention group, 95/103 (92.2%) of the patients reported to have found the videos helpful, 96/103 (93.2%) of the patients found that the videos helped them pass time while waiting for their appointment, and 78/103 (75.7%) of the patients were interested in watching MSK self-care videos in the future. In the control group, 88/102 (86.2%) of the patients found the videos helpful, 92/102 (90.1%) of the patients agreed that the videos helped them pass time, and 80/102 (78.4%) of the patients wanted to watch MSK self-care videos in the future. No adverse events were reported in either group throughout the sessions. Patients’ testimonials regarding videos were positive. One patient stated:

I never realized that a person could find pressure points on their body to help relieve issues like nausea and GI discomfort. These videos were very informative!

**Table 2:** Patient-rated experiences.

Survey questions	Intervention group		Control group	
	Yes, n (%)	No, n (%)	Yes, n (%)	No, n (%)
Were these videos helpful to you?	95 (92)	8 (8)	88 (86)	14 (14)
Did these videos help you pass time during your visit today?	96 (93)	7 (7)	92 (90)	10 (10)
In the future, would you like the option to watch the Memorial Sloan Kettering Cancer Center’s videos while waiting for your appointments?	78 (76)	25 (24)	80 (78)	24 (22)

Similarly, another patient reported that the video “helps to pass time while waiting for treatment. Helpful tips to decrease anxiety and stress.” Interest in viewing more interventional videos in the future was also expressed:

Very helpful, I would like it if there were links to video showing some of the meditation or yoga techniques.

## Discussion

### Principal Findings

Prolonged wait times in chemotherapy waiting rooms account for many of the negative experiences associated with cancer treatments. Anticipating the awaited treatment can induce debilitating symptoms such as anxiety and stress. Although prior research and QI efforts have focused on shortening treatment wait times, limited investigation has been done on how to enhance overall patient experiences in chemotherapy waiting rooms. This study showed that we could utilize an integrative medicine app effectively and safely to improve patients’ waiting room experience.

In this study, we demonstrated that the use of an integrative medicine app to deliver guided acupressure and mindfulness meditation is feasible and beneficial in chemotherapy waiting rooms. We found a significantly higher reduction in stress and anxiety levels in the group exposed to the acupressure and meditation video when compared with the control group. In addition, we found a significant reduction in pain compared with baseline in the intervention group, whereas there was no significant change in pain in the control group. Although this study showed that our intervention videos did not improve nausea, this may be because of the fact that our participants demonstrated a very low median baseline nausea score (0.8 out of 10) to start with, leaving little room for improvement.

The efficacy of acupressure in relieving chemotherapy-induced stress and anxiety, as shown in this study, is consistent with findings in previous studies, which showed that different forms of acupressure significantly reduced pretreatment anxiety and chemotherapy-induced nausea [24,25]. A previous QI study found that providing patients with guided meditation delivered through iPads decreased average distress levels by 46% (*P*<.01) in patients undergoing chemotherapy [26]. Another clinical trial also found that mindfulness interventions significantly lowered blood pressure, heart rate, oxygen saturation, and perceived stress in women undergoing breast biopsies [27]. A recent study in 108 cancer patients also showed that a single-session mindfulness practice significantly reduced anxiety levels and lowered heart rates in patients undergoing positron emission tomography-computed tomography scans [28].

However, this is the first study to utilize an app to educate patients on using acupressure and mindfulness meditation to achieve the same outcomes of significantly reducing patient anxiety and stress. Our results further demonstrated the effectiveness and feasibility of extending this therapy to chemotherapy waiting rooms to enhance patient experiences while awaiting treatment. In addition, the overall feedback we received from participants about their experiences was very positive, with a majority of them expressing that the videos were helpful, helped them pass time, and that they wanted further access to such videos.

Although we were not surprised that the intervention group demonstrated a significant reduction in stress and anxiety, it is interesting to note that the control group also demonstrated a reduction in stress and anxiety levels, suggesting that symptomatic relief may also be accomplished by certain distractions. This finding suggests that the integrative medicine app can serve as a platform to educate patients about available integrative cancer therapies and also aid in alleviating symptoms. It is important to note that our intervention videos resulted in greater symptomatic relief, indicative of effects extending beyond distraction alone. Overall, this study demonstrates that an integrative medicine app could be an invaluable tool for enhancing the overall chemotherapy waiting room experience. As adverse symptoms are often responsible for poor chemotherapy tolerance, the efficacy of these technological apps in alleviating symptoms through delivering self-care techniques can potentially improve treatment tolerance.

There are a few limitations to this study. It was a single-center study, and we excluded non-English speaking patients. In addition, patients could have more relief from their symptoms if they watched the video more than once. This study is further limited by the exclusion of patients who were scheduled to have their first chemotherapy session. As these patients are often already overwhelmed, we assumed that their baseline symptoms would be heightened and the effectiveness of the intervention would not be accurately assessed. Finally, our assessment focused on the immediate effect of the intervention on symptoms. We do not have any information on the effect of our intervention, if any, in the long term (ie, if patients implemented the techniques they learned through the app at subsequent time points and what the effect of these may have been).

To the best of our knowledge, this is the first study to explore the use of self-care apps to improve patients’ waiting room experiences. Previous studies have investigated the use of mobile apps in patient care coordination and clinical practices but not in the chemotherapy waiting room [29,30].

This study showed the feasibility and effectiveness of an integrative medicine app in waiting rooms and provided the foundation for future exploration of expanding the use of technological interventions in this setting. To improve the integrative medicine app experience, we could provide devices available at check-in, make the app accessible on personal devices, and provide direct links to self-care videos as well as the MSK integrative medicine Web page for additional resources. Future research could focus on expanding this platform in other waiting room settings to improve overall patient treatment experiences.

### Conclusions

Providing self-care tools through an integrative medicine app in the waiting room improved patients’ experiences and reduced overall anticipation-induced anxiety and stress levels. Future research could focus on expanding this platform to other settings to improve patient treatment experiences and increase awareness of integrative medicine.

## Abbreviations

MSK: Memorial Sloan Kettering Cancer Center
QI: quality improvement
